# Extensive primary production promoted the recovery of the Ediacaran Shuram excursion

**DOI:** 10.1038/s41467-021-27812-5

**Published:** 2022-01-10

**Authors:** Fuencisla Cañadas, Dominic Papineau, Melanie J. Leng, Chao Li

**Affiliations:** 1 Department of Earth Sciences, University College London, London, UK; 2 London Centre for Nanotechnology, University College London, London, UK; 3 Centre for Planetary Sciences, University College London & Birkbeck College London, London, UK; 4 State Key Laboratory of Biogeology and Environmental Geology, China University of Geosciences, Wuhan, China; 5 National Environmental Isotope Facility, British Geological Survey, Nottingham, UK; 6 School of Biosciences, University of Nottingham, Loughborough, UK; 7 Present Address: Centre for Astrobiology (CAB, CSIC-INTA), Madrid, Spain

**Keywords:** Carbon cycle, Geochemistry, Precambrian geology

## Abstract

Member IV of the Ediacaran Doushantuo Formation records the recovery from the most negative carbon isotope excursion in Earth history. However, the main biogeochemical controls that ultimately drove this recovery have yet to be elucidated. Here, we report new carbon and nitrogen isotope and concentration data from the Nanhua Basin (South China), where δ^13^C values of carbonates (δ^13^C_carb_) rise from − 7‰ to −1‰ and δ^15^N values decrease from +5.4‰ to +2.3‰. These trends are proposed to arise from a new equilibrium in the C and N cycles where primary production overcomes secondary production as the main source of organic matter in sediments. The enhanced primary production is supported by the coexisting Raman spectral data, which reveal a systematic difference in kerogen structure between depositional environments. Our new observations point to the variable dominance of distinct microbial communities in the late Ediacaran ecosystems, and suggest that blooms of oxygenic phototrophs modulated the recovery from the most negative δ^13^C_carb_ excursion in Earth history.

## Abstract (web summary)

Variable dominance of distinct microbial communities during the late Ediacaran, recorded in C and N cycles perturbations and in Raman structural heterogeneities of organic matter, modulated the recovery from the most negative δ^13^C_carb_ excursion in Earth’s history.

## Introduction

The Ediacaran Shuram excursion (c. 570 – c. 551 Ma) records a significant C cycle perturbation in Earth history with unusually light carbonate carbon isotope values (δ^13^C_carb_) down to −12‰ and a characteristic lack of covariation between carbonate and sedimentary organic carbon isotope trends^1,2^. This isotope excursion was first described from the Shuram Formation in Oman^3^. Afterwards, correlative studies from Australia, California, and South China^1,4,5^ showed this excursion was a global phenomenon. Although intensive studies have been conducted, the mechanisms driving the origin and termination of the Shuram excursion remain debated (see reviews in refs. ^6–8^).

There are various hypotheses proposed to explain the origin of the Shuram excursion, and these can be summarised in two principal groups. The first group proposes that the excursion results from secondary, post-depositional alteration during burial diagenesis, along with meteoric water or authigenic carbonate precipitation^9–11^. The second group, however, suggests that the excursion is a primary depositional carbon isotope signature related to the oxidation of massive ^13^C−depleted dissolved organic matter (DOC)^12^ or other types of organic carbon pools^13,14^ during a globally synchronous ocean oxygenation event^3,5^. In the first case, the diagenetic overprint of the isotopic signal would have required sediments to be chemically preconditioned so that local processes produce the same signal globally. The second case would imply the existence of a massive DOC pool in which partial or complete oxidation would have required abundant oxidants. Both hypotheses are controversial with equivocal solutions. However, they may not necessarily be mutually exclusive if some C-recycling processes started in the water column and continued during diagenesis.

While it is possible that the Shuram excursion was a consequence of diagenesis, increasing evidence support that the excursion can also represent the oxidation of the marine organic carbon pool. The abundance of cyanobacterial-like microfossils in the black shales of the underlying Member II^15^, combined with ^13^C−enriched carbonate^16^ and widespread phosphorite deposits in the Doushantuo Fm.^15,17,18^ suggest the existence of elevated concentrations of organic matter from primary producers in the environment. In addition, if the DOC oxidation drove the excursion, it is expected that there would be a close relationship between the oxygenation of the water column and the Shuram excursion. In support of this, shallow oxic conditions are described from different environments^5,19–21^. In these studies, Fe speciation and S isotopes are used to conclude that organic-rich shales of Member IV were deposited in euxinic environments. However, these studies also report high δ^238^U, δ^98^Mo, and Mo concentrations that suggest extensive ocean oxygenation during the deposition of the Member IV. Lastly, recent quantitative models propose a sufficient and realistic oxidant budget for heterogeneous or partial oxidation of the DOC occurring only on shelf areas of the global Ediacaran oceans^22^, or the total DOC exhaustion when surplus oxidant is generated through bacterial reduction of sulfate weathered from evaporite deposits^8^.

The recovery from the Shuram excursion to pre-excursion δ^13^C_carb_ values has been previously associated with the complete oxidation of a DOC pool^12^, decreasing input of weathered detrital organic carbon^13^, decreasing expelled hydrocarbons from sedimentary organic matter^14^, or reduced local DOC availability via persistent consumption of reduced carbon at the shelfs^7^. In the South China Nanhua Basin, the recovery from the Shuram excursion is recorded in Member IV of the Doushantuo Formation (ca. 560 − 551 Ma). This member consists of organic−rich shales of variable thickness (1 − 30 m) deposited during a sea-level highstand with total organic carbon (TOC) contents up to 15%^23^.

Here, we use new bulk C and N isotope and Raman data from Member IV shales in six sections that span shallow to deep environments in the Nanhua Basin to understand the biogeochemistry in the basin and its relationship to the Shuram excursion, specifically with the recovery to pre-excursion δ^13^C_carb_ values. Future studies should focus on sedimentological and mineralogical observation to further explore the diagenetic phenomena associated with the oxidation of organic matter during this critical Neoproterozoic period^24^.

## Results

### Chemostratigraphic profiles

**Fig. 1:**
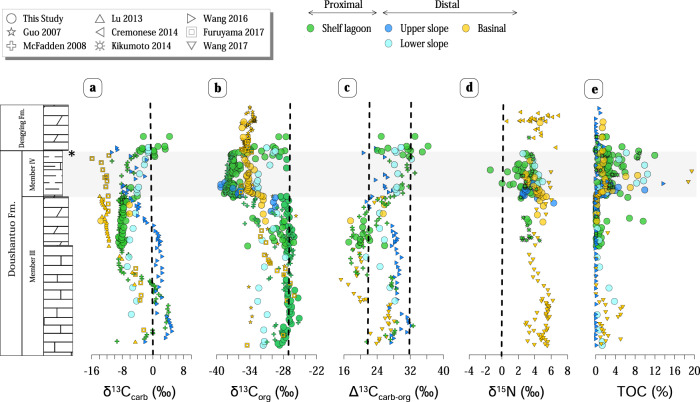
New chemostratigraphic columns of carbon and nitrogen isotope compositions in Doushantuo Member IV. **a** Carbonate carbon isotopes. **b** Organic carbon isotopes. **c** Carbonate and organic carbon isotopes difference. **d** Sedimentary nitrogen isotopes. **e** Total organic carbon content. Data are classified by depositional environments and, where applicable, shown in comparison with published data^5,26,28–30,68^. *U − Pb age of 551.1 ± 0.7 Ma^69^. Vertical dashed lines from left to right show Peedee belemnite, isotopic fractionation imparted to biomass by primary producers typically around −27‰, photosynthetic range 22–32‰ based on compilations of Δ^13^C_carb-org_ throughout the Phanerozoic^70^, and lastly δ^15^N for atmospheric N_2_.

C and N concentration and isotope data from six shelf−to−basin depositional environments of the Nanhua Basin are illustrated in Fig. 1, summarised in Table 1 and detailed in Table S1. Table 1 shows stratigraphic end-members of Member IV (described in Supplementary Information), representing the chemo-stratigraphic trends for the evolution of the measured C and N geochemical parameters. Proximal sections include Xiangerwan, Zhimaping, and Qinglinkou, which are located in the shelf lagoon paleo-environment^25^. Distal sections include Taoying (upper slope), Xiajiaomeng (lower slope), and Fengtan (basin)^25^ (see Fig. S1a–c). The proposed stratigraphic correlation of Member IV of these sections is based on new and published data from the studied and nearby sections that constrain the lateral variability and isotopic expression of the Shuram excursion in South China^5,26–28^. More details on the geological context of the studied sections can be found in Supplementary Information. In addition, potential postdepositional alteration of isotope signatures was evaluated based on δ^13^C_org_-TOC, TOC-TN, TN-δ^15^N, and Mn/Sr-δ^13^C_carb_ plots and resulted in no significant alteration detected in the studied samples (see Supplementary Information).

**Table 1:** Summary of geochemical results.

		δ^13^C_carb_ (‰)		δ^13^C_org_ (‰)		δ^15^N (‰)		TOC (%)		*n*
Environment	Section	Base	Top	Base	Top	Base	Top	Base	Top	
Shelf Lagoon	Xiangerwan	**−4.1**	**−0.6**	−37.7	−36.9	+4.4	+3.6	2.7	4.6	7
	Zhimaping	**−7.1**	**−0.2**	−38.6	−34.6	+3.7	+2.9	5.0	1.3	51
	Qinglinkou	−5.8	**−3.6**	−37.0	**−27.7**	+3.3	+2.3	0.3	9.6	15
Slope	Taoying	−4.3^*^	−11.3^*^	−35.7	−38.5	+2.5	+3.7	1.1	1.7	10
	Xiajiaomeng	**−3.7**	−1.2	−32.8	−29.8	+4.3	+2.6	8.3	6.2	6
Basin	Fengtan	−11.5^**^	−15.9^**^	−32.0	−34.4	+5.4	+3.4	0.3	0.9	21

Data show end-member values of Member IV (base and top). In sections where data at the base and top of Member IV were not available, data from the Member III-Member IV and Member IV-Dengying Fm. boundaries were used and are expressed in bold. (*) represents data from an analogous upper slope environment section (Siduping^30^). (**) represents data from the Fengtan section^28^. *n*= number of samples.

The carbon isotope composition of carbonates and organic matter (δ^13^C_carb_ and δ^13^C_org_) from the proximal sections preserve the most similar signal to the Shuram excursion in Oman^3^. In this environment, values of δ^13^C_carb_ from the uppermost part of Member III to the Member IV-Dengying Fm. boundary exhibit a trend that gradually shifts from −7.1‰ to −0.2‰ (Fig. 1a). Similarly, values of δ^13^C_org_ of the same interval present a shift from ∼−28‰ to −38.6‰ and recover again to ∼−28‰ at the Member IV-Dengying Fm. boundary (Fig. 1b). These characteristic trends are consistent with previously reported data from nearby outcrop samples^2,5,26,29^. On the other hand, the distal sections present values of δ^13^C_org_ from −32.8‰ to −38.5‰ and δ^13^C_carb_ values from −4.3‰^30^ to −15.9‰^28^ upward section. In contrast to the recovery trend in the proximal sections, these isotope trends for distal sections vary toward more ^13^C-depletions at the top of Member IV. The exception to this is found in the Xiajiaomeng section, located in the distal lower slope environment and presents the same δ^13^C patterns as the proximal sections with increasing δ^13^C_org_ from −32.8‰ to −29.8‰ and δ^13^C_carb_ values from −3.7‰ to −1.2‰ upward section.

Previous late Ediacaran δ^15^N data from the studied area are scarce and have been reported from only two sections in proximal environments^26,31^, and one in distal environments^30^, and have a range in values between +5.4‰ and +2.3‰ (*n* = 83) (Fig. 1d) towards the top of the Doushantuo Fm. Results from this study are thus consistent with previous data. However, they present a new and complete shelf−to−basin N profile, exclusively spanning Member IV. Towards the top of Member IV, most data show similar decreasing trends except the Taoying section, which instead presents increasing δ^15^N values from +2.5‰ to +3.7‰. δ^15^N variations from the base to the top of Member IV in the distal environments range from +5.4‰ to +2.6‰. In the proximal area, however, Zhimaping shows more significant δ^15^N variations with a characteristic trend formed by two different cycles upward section separated by sharp boundaries with values between +3.7‰ to 0‰ and from +3.6‰ to –1.4‰ respectively.

**Fig. 2:**
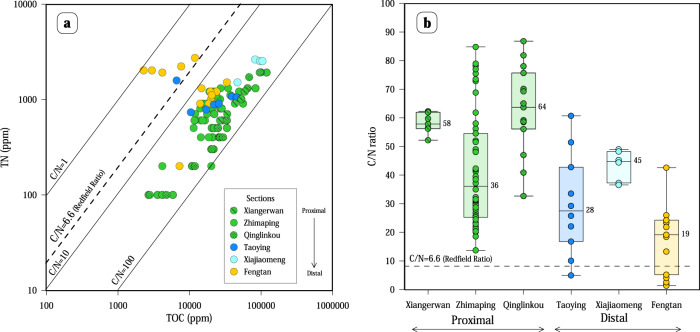
Measured C and N abundances in the different depositional environments. **a** C/N ratio presented by stratigraphic sections. The dashed line represents the Redfield ratio (C/N = 106/16). **b** Statistical distribution of C/N ratios from shallow to deep environments showing the full range of ratios (ticked lines), the mean (lines with a number), and one standard deviation distribution (box).

Similarly, reported Precambrian C/N ratio data are limited, and most of these data have a high C/N ratio^32^. Measured C/N ratios of Member IV black shales also markedly exceed the Redfield ratio of 6.6^33^, as shown in Fig. 2, with 94% of values between 10 and 90 (*n* = 104). Sections from the proximal area collectively show the highest C/N ratios, close to 90. Comparably, results from distal sections (Taoying and Fengtan) show the lowest C/N ratios, significantly below the Redfield Ratio, with values as low as 1.3. The statistical C/N distribution between environments also reflects higher mean values in the proximal sections (*n* = 73) compare to the distal sections (*n* = 31), which show the lowest mean values in the basin.

### Raman spectral characteristics of organic matter

**Fig. 3:**
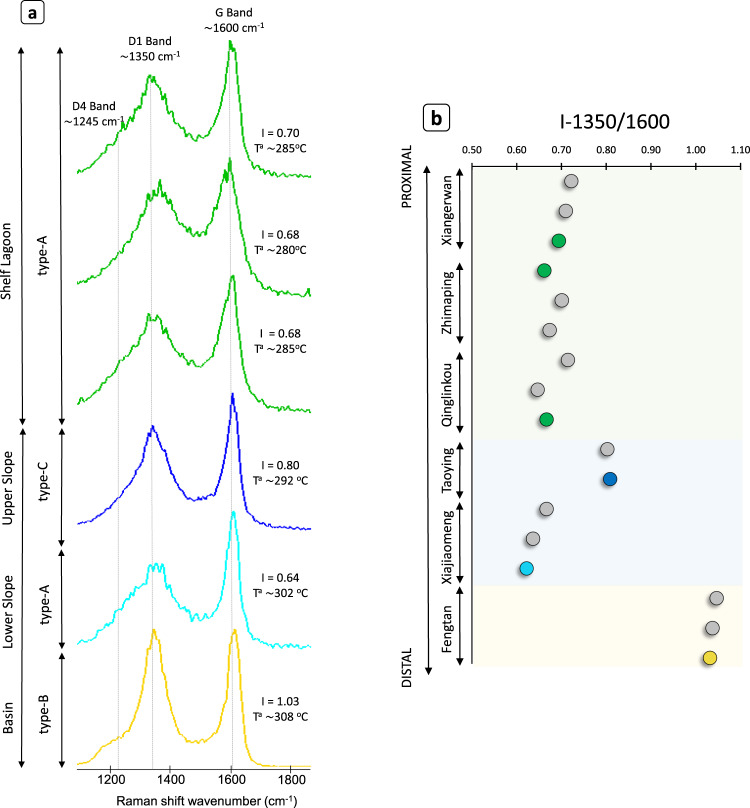
Raman spectra of organic matter in Doushantuo Member IV. **a** Raman spectra are organized by depositional environments and show the disordered but variable structure of the different kerogen types proposed (A, B and C). The two broad bands at ∼1350 cm^−1^ and ∼1600 cm^−1^ are used to calculate peak metamorphic temperatures and yield an intensity ratio defined as I-1350/1600 (I in this figure). D4 band is a secondary band that shows a well-developed shoulder in the basin sample. **b** Compilation of I-1350/1600 results from seventeen samples of the studied sections showing the distribution of the calculated intensity ratio along the basin. The colored circles represent samples shown in **a**.

Raman spectra of organic matter in the studied samples show the typical features of disordered organic matter, with two broad bands at ∼1350 cm^−1^ (or D1 band) and ∼1600 cm^−1^ (G band). These characteristic D1 and G bands, together with their intensity ratio, defined as I-1350/1600, allow the identification of different predominant types of kerogen structures shown in Fig. 3. For example, kerogen structure type-A predominates in proximal and lower slope sections, and it is characterized by a wide D1 band, a resolvable shoulder of the D4 band at ∼1245 cm^−1^, and a more intense and narrower G band with a I-1350/I-1600 between 0.64 and 0.70. Kerogen structure type-B dominates in the basinal environment (Fengtan) and is characterized by narrower D1 and G bands with similar intensities and the I-1350/I-1600 1.03 (Fig. 3). In addition, the upper slope area shows a mixed type-C kerogen structure characterized by a more intense G band, similar to type-A, and narrower D1 and G bands, similar to type-B and with an intermediate I-1350/I-1600 value of 0.8 (Fig. 3). Importantly, these spectra of organic matter are used to estimate peak metamorphic temperatures (Supplementary Information; Table S2) with results between 280–289 ^o^C in the proximal sections and between 300–309 ^o^C in the distal sections, thereby confirming the authigenecity of organic matter.

### Nutrient availability in Late Ediacaran oceans

Phosphorous and nitrogen are two limiting nutrients that play a significant role in regulating the primary production of organic matter, so understanding their availability in the Ediacaran oceans is essential to evaluate the impact of organic matter production on the Shuram excursion recovery. The occurrence of widespread phosphorite deposits in Member IV^34,35^ and blooms of oxygenic photosynthesis during the Ediacaran^15^, and notably similar at both ends of the Proterozoic^36^, point to the high availability of dissolved phosphorous that led to eutrophication in the water column with high primary productivity in surface waters and high organic carbon fluxes into sediments.

Nitrogen is the other major control for marine primary production. In general, the balance in the N input-output processes will determine the δ^15^N composition of oceanic N, where N_2_-fixation and denitrification are the major processes^37^. N enters the ocean mainly through the biological fixation of atmospheric N_2,_ while denitrification is the main pathway of N loss from the ocean^38^. Both processes present characteristic isotopic fractionations (*ε* = δ^15^N_product_ – δ^15^N_reactant_): N-fixation ∼0 to −4‰^38^, and denitrification between +15‰ and +30‰^39^. Our results reveal homogeneous spatial δ^15^N variations with values between +5.4‰ to +2‰, comparable with modern shelf sediments. In modern oceans, nitrate is partially reduced by microbial denitrification into N_2_ or N_2_O with significant isotopic fractionation, resulting in ^15^N-rich nitrate, which is then assimilated into biomass^38^. In other words, denitrification or assimilation processes exceed the supply of nitrate into the modern ocean. Thus, within the modern δ^15^N values range, the shift from the base to the top of Member IV reflects a dynamic environmental scenario from initial denitrification or nitrate assimilation dominance towards a new ecological state evaluated below.

Two principal mechanisms can account for the decrease of δ^15^N: increasing nitrate availability and/or enhanced N-fixation. In the first case, although this scenario is compatible with gradual oxidation of the Ediacaran oceans^3,5^, prolonged anoxic conditions in the deep ocean during the Ediacaran^25,40^ likely contributed to extensive denitrification that removed nitrate from seawater. Thus, nitrate unlikely exceeded denitrification, which does not support a model of increased nitrate availability. Alternatively, high P conditions, likely promoted by combined continental weathering^41^ and P remineralization^42^, would have led to active biological N_2_-fixation to overcome the N loss due to denitrification. This is because dissolved N is tightly correlated with dissolved P in open ocean water, with an N/P ratio of 16/1^33^, and to maintain the ratio relatively constant, instantaneous changes in the nitrate reservoir through denitrification would have led to increased N_2_-fixation^43^ to return the nitrate reservoir to its initial size, closer to the Redfield ratio. In addition, high phosphorus availability in the proximal area, supported by the occurrence of widespread phosphorite deposits, could also explain the lowest δ^15^N values reached in the Zhimaping section, as low as –1.4‰, as areas with high phosphorus content would have experienced more intense N_2_-fixation to maintain the N/P ratio constant. Hence, the general decrease of δ^15^N values in the Member IV is best explained as a new equilibrium in the N cycle, whereby N_2_-fixation becomes dominant over denitrification as the predominant biological process modulating sedimentary δ^15^N values.

### Extensive primary production drove the recovery of the Shuram excursion

One of the principal characteristics of the Shuram excursion is the lack of covariation between carbonate and sedimentary organic carbon δ^13^C trends. Covariation between coeval δ^13^C_carb_ and δ^13^C_org_ isotope records has been used to assess changes in the ancient dissolved inorganic carbon (DIC) pool. This is because, under the conventional understanding of the carbon cycle, δ^13^C of organic and carbonate carbon is derived from the same marine DIC reservoir and covary accordingly^12^. Therefore, coupled δ^13^C_carb_ and δ^13^C_org_ signatures reflect primary δ^13^C signatures of seawater while decoupled δ^13^C_carb_ and δ^13^C_org_ signatures are interpreted as evidence for diagenetic alteration in sediments^9–11^ or oxidation of organic matter in the water column^3,5,12^.

Paired data sets of δ^13^C_carb_ and δ^13^C_org_ in this study are mostly decoupled during the Shuram excursion and exhibit a coupled trend only in the uppermost part of Member IV. This is consistent with previous data reported in South China^5,26^ and shows the same pattern as the Shuram excursion in Oman^3^. Although it cannot be entirely ruled out that the excursion was a consequence of diagenesis, in the context of this study, the negative δ^13^C_carb_ excursions are interpreted as the result of the remineralization of a massive ^13^C−depleted DOC pool^12^. The turning point to coupled and more positive trends would reflect a transition towards a new environmental state proposed to be dominated by oxygenic photosynthetic primary producers under extensive bloom conditions.

**Fig. 4:**
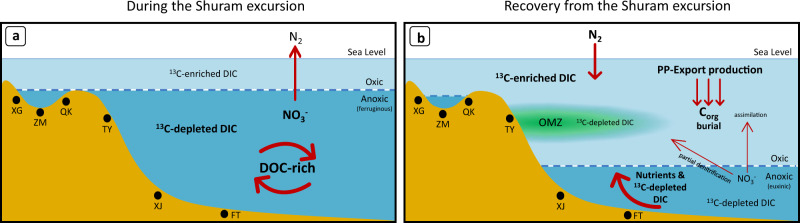
Schematic diagram showing the biogeochemical evolution of the late Ediacaran ocean. **a** During the Shuram excursion, denitrification was the dominant N biogeochemical process. Recycling and remineralization of DOC by heterotrophic organisms led to a ^13^C−depleted rich DIC pool. Anoxic waters were predominantly ferruginous^23,34^. **b** During the recovery from the Shuram excursion N_2_-fixation became the main N biogeochemical process. Extensive PP in shallow waters favored the increase of organic carbon fluxes towards deep waters and the ^13^C−enriched DIC budget, which counteracted the ^13^C−depleted DIC pool and led to positive shifts in δ^13^C_carb_ and δ^13^C_org_. Anoxic waters were predominantly euxinic^23^. XG Xiangerwan, ZM Zhimaping, QK Qinglinkou, TY Taoying, XJ Xiajiaomeng, FT Fengtan. DOC dissolved organic carbon, DIC dissolved inorganic carbon, PP Primary production, OMZ Oxygen Minimum Zone. Text in bold represents the dominant processes/fractions.

The new proposed environmental state is illustrated in Fig. 4, which represents a late Ediacaran C and N cycle evolution during the Shuram excursion and the recovery to pre-excursion values. Before the recovery, Fig. 4a, the environmental scenario was characterized by denitrification as the principal N metabolic pathway and heterotrophic remineralization of the existing DOC that led to ^13^C−depleted rich DIC pool and, thus, low δ^13^C_carb_. However, during the recovery (Fig. 4b), extensive primary productivity in shallow waters led to increased export production and extensive organic carbon burial. Consequently, the ^13^C−enriched DIC budget was considerably increased and eventually counteracted the existing ^13^C−depleted DIC, probably aided by decreasing DOC oxidation suggested previously^3,7^, and drove positive shifts in both δ^13^C_carb_ and δ^13^C_org_, thus the recovery from the Shuram excursion. In this latter scenario, N_2_-fixation became the primary N metabolism in the N biogeochemical cycle that, together with phosphorous availability mentioned above, led to extensive primary production in the surface.

The dominance of primary over secondary production has also been proposed for the contemporaneous Shuram Formation in Oman, where preserved organic biomarkers reveal multiple sources of syngenetic organic matter with more significant contributions from bacterial than algal biomass during the recovery of the Shuram excursion^44^. In addition, the δ^15^N shift toward lower values, and thus dominance of N_2_-fixation metabolism, would have promoted the ecological advantage to N_2_-fixing cyanobacteria relative to eukaryotic algae, which lack the capacity for N_2_-fixation and instead preferentially assimilate nitrate^45^.

The variable dominance of distinct microbial communities can be tested from the C/N data. Laboratory experiments reveal that during early and maximum blooms, dominated by primary producers such as cyanobacteria, C/N ratios tend to increase above the Redfield ratio to a maximum because rates of total N uptake exceed those of regeneration^46^. However, during postbloom periods, dominated by consumer interactions, C/N ratio tends to decrease close to the Redfield ratio because of the degradation of low molecular weight organic carbon^47^. The C/N ratios show a heterogeneous distribution between the studied sections (Fig. 2), and are consistent with the dominance of primary or secondary producers in the different environments. For example, values near the Redfield ratio in the distal environments (C/N between 1.3 and 26) suggest the dominance of secondary production. However, values higher than the Redfield ratio in proximal environments (C/N between 20 and 85) are consistent with those reported from this period in South China^26,30^ and elsewhere^31^ and represent periods of blooms of primary production.

### Source mixing between primary and secondary organic matter

The new photosynthetic C produced near the surface may have largely coexisted with older and recalcitrant DOC principally stored in deep waters but ultimately brought to shallow depths via upwelling^12,48^. The heterogenous δ^13^C trends in the basin reflect a source mixing between autotrophic and heterotrophic pools and describe a transient C-cycle characterized by a longer C residence time during the late Ediacaran than today’s ocean-atmosphere system, which is 10^5^ years. Thus, it is suggested that a heterogeneous decrease of the DOC pool size after extensive remineralization into ^13^C−depleted carbonate would have led to the δ^13^C trends heterogeneity along the basin, as previously proposed^7^.

Three different factors may have accounted for this heterogenous DOC pool size reduction. First, the presence of anoxic-euxinic conditions in the mid-slope and deep basinal environments^25,40^ allowed anaerobic degradation of organic matter to dominate the production of ^13^C−depleted DIC buried in carbonate precipitates. Second, the chemically resistant nature of the refractory DOC in the deep ocean, which accounts for 72% of the total organic carbon in the modern oceans^49^, led to less efficient microbial oxidation. This is because the refractory fraction consists of high molecular weight and structurally complex compounds rich in carbon resistant to microbial oxidation and degradation^50,51^. Third, upwelling during transgression would have delivered ^13^C−depleted bicarbonate together with regenerated nutrients from the deep sea to the photic zone, which would fuel primary productivity and trigger a turnover of ^13^C−depleted mid-slope and basinal waters.

Extensive primary production in shelf environments would lead to a sulfidic oxygen minimum zone (OMZ) immediately below the productivity zone^23,52^, which would favor the preservation of organic matter. This scenario explains why the distal lower slope section (Xiajiaomeng), interpreted to be located immediately below the OMZ, presents a carbon isotope trend similar to the proximal environment, characterized by high rates of organic carbon burial. An OMZ also helps to explain why only the Taoying section, located in the upper slope, shows an increase in δ^15^N values likely due to anammox and heterotrophic denitrification, which led to substantial N-loss. This is because in OMZs, and other low oxygen systems, nitrogen loss by denitrification or assimilation exceeds nitrogen supply in the zone, and the remaining nitrate will become progressively enriched in ^15^N(^53^). Any of the three factors mentioned above, or a combination of them, imply a dynamic situation where DOC is continuously produced and recycled, in which case the different δ^13^C_carb_ and δ^13^C_org_ trends represent higher primary than secondary production rates, allowing the DOC pool to wax and wane.

### Structural heterogeneity of organic matter

In order to independently confirm the occurrence of different sources of biomass in the Nanhua Basin, Raman spectra of organic matter are investigated. The Raman spectra of organic matter in the studied samples reflect the presence of different types of kerogen structure (type-A, type-B, and type-C; Fig. 3). One reason that may account for different kerogen structures is the metamorphic grade that affected the organic matter. However, the peak temperature analysis (see Supplementary Information; Table S2) shows that all the evaluated samples are below or near the base of the greenschist metamorphic grade, around 280−308 ^o^C in proximal and distal environments, respectively. In addition, there is no correlation between temperature and spectral characteristics in the studied samples (Fig. 3a). For example, spectra of organic matter in the shelf lagoon and lower slope have a similar shape and I-1350/1600 values but differ by ∼15–20 ^o^C in their peak metamorphic temperature. Similarly, the Raman spectra of organic matter between the lower slope and basin show similar peak metamorphic temperatures between 302 and 308 ^o^C, but remarkably different shapes and I-1350/1600 values. Therefore, these observations imply that all these rocks were exposed to the same temperature and pressure regime of regional metamorphism, and thus, the observed different kerogen structures are not associated with metamorphic temperature variations along the basin.

An alternative interpretation for kerogen variability could be the differential degradation of organic matter in the column water. In marine environments, the two principal factors that may impact organic matter degradation are temperature and redox conditions. Paleotemperatures inferred from isotope compositions (δ^18^O and δ^30^Si) in chert have been used to estimate the late Neoproterozoic seawater temperature to be around 25–28 ^o^C^54,55^. This range is only slightly higher than modern ocean surface water, which is about 20 ^o^C. Thus, the kinetics of thermal mechanisms affecting organic matter degradation (aerobic respiration, fermentation, etc.) likely worked similarly to today. Hence, redox conditions might be a major factor affecting organic matter degradation. Redox conditions in the Nanhua Basin have been studied and inferred from multiple geochemical proxies. For example, sulfur isotope composition of pyrite and iron speciation in different proximal and distal sections suggested prevailing ferruginous/euxinic deep waters during the late Ediacaran^5,23,25^. Likewise, high uranium and negative molybdenum isotope trends measured in the same environments were interpreted as extensive ocean oxygenation during the deposition of Member IV black shales^20,21^.

In the scenario that only a single type of organic matter was present and preserved, the basin-wide stratified redox conditions would have allowed a relatively homogeneous chemical degradation of biological organic matter throughout the basin. Furthermore, the likely similar sinking path in the water column and the eventual anoxic/euxinic layer at the sea bottom and sediment-water interface would have caused organic matter sequentially to lose the same functional groups and undergo aromatization during degradation irrespectively of the environment in it was deposited. Consequently, a similar Raman spectra fingerprint could be expected, regardless of its position in the basin. However, Raman results described in this study show well-differentiated spectra that require alternative explanations.

A plausible solution for different kerogen structures could be the variable composition of organic matter associated with various biomass sources, as previously argued^56^. In recent studies, heterogeneities in the Raman spectral I-1350/1600 parameter of organic matter in microfossils have been interpreted to be associated with the presence of organic compounds derived from different sources. This is because the structural ordering of organic matter depends on the original composition of the biological precursor^57–59^. This heterogeneity is remarkably evident among the studied sections with I-1350/1600 values between 0.62 and 1.05 (Fig. 3b and Table S2, SI) and with a remarkable distribution easily correlated with the different environments. In addition, specific characteristics of Raman spectra also may point to various biomass sources. For example, the D4 band is present in all samples but is exceptionally well developed in the most distal section (Fig. 3 and Fig. S4b), where it becomes a distinctive feature. The D4 band (at ∼1245 cm^−1^) often appears as a broad shoulder of the D1 band^60^. The most common interpretations of the origin of the D4 band focus on the increase of structures with sp^2^-sp^3^ hybridization and C-C or C = C bonds^61^. It is generally absent in purely graphitic or highly carbonized materials but present in less mature OM where the presence of C-C on aromatic rings provides robust chemical structures that may prevent degradation^62^.

As mentioned in the previous section, refractory DOC is formed by structurally specific compounds rich in C-C and C = C bonds resistant to microbial oxidation and degradation^50,51^. This observation allows interpreting that kerogen structure type-B, with distinctive D4 band, represents resistant organic matter rich in C-C and C = C bonds, thus refractory DOC that would have persisted in the water column. A widely accepted mechanism links the chemical diversity of refractory DOC to microbial diversity^63,64^. These studies identified associations between the production of refractory DOC with specific bacterial and archaeal groups whereby these organisms altered the molecular structure of DOM and made it resistant to further degradation, preserving fixed carbon in the ocean. It was also observed that the rate of formation of refractory DOC was dependent on the rate of microbial activity^63^. Therefore, it is suggested that kerogen structure type-B represents refractory DOC that dominated in the distal environment and was partly sourced by microbial activity or heterotrophic remineralization. Accordingly, kerogen structure type-A is suggested to represent photosynthetic production based on its predominance in shallow environments. In the surface ocean, the photosynthesis of organic molecules from CO_2_ by phytoplankton is the source of most of the ocean’s labile DOC^65^, which is compositionally distinct from refractory DOC. For example, proteins, carbohydrates and aliphatic materials are enriched in labile DOC relative to the signatures of the deep ocean, which is rich in resistant aromatic compounds^66^. Thus, the less pronounced D4 band shoulder, thus less C-C or C = C bonds, in proximal samples could represent fewer aromatic compounds and, therefore, labile organic matter.

However, this Raman spectra evaluation, considered alone, cannot be used to distinguish primary from secondary productivity alone, but along with C and N isotope data, their combination shows promising results. The described Raman characteristics of the organic matter from the basin have a distribution remarkably similar to the C isotope trends described in this study. Specifically, the shelf lagoon and lower slope sections, with kerogen structure denominated as type-A and I-1350/1600 values between 0.62-0.72, are characterized by δ^13^C recovery trends. In contrast, the basin section, which hosts kerogen of type-B and I-1350/1600 values around 1.05, shows a δ^13^C trend towards lower values. The correlation between kerogen types and δ^13^C trends further indicates that kerogen type-A was the most dominant type in the Nanhua Basin and represents organic matter sourced from primary production. If so, dominant kerogen type-B sourced from heterotrophic production during the Shuram excursion was overcome by kerogen type-A sourced from extensive primary production, pointing to a new environmental ^13^C−enriched DIC scenario that eventually promoted the recovery from the Shuram excursion.

High primary production in Member IV of the Doushantuo Formation is reflected by the occurrence of organic-rich shales and phosphorite deposits. This increased primary production was likely stimulated by phosphate availability and the consequent increased N_2_-fixation and CO_2_-fixation. The enhanced primary production is consistent with recently modelled organic carbon fluxes during the Ediacaran, which predicts a higher proportion of organic carbon burial than today^67^. The ensuing recovery to near-zero δ^13^C_carb_ values was modulated by primary producers, who fix both C and N. This new biological pump would have caused increased exports of nutrients and organic carbon to sediments. The identification of different types of organic matter by Raman spectroscopy lends independent support for the presence of various sources of biomass, which is most parsimoniously interpreted as primary and secondary given identical metamorphic grades. Hence, the dominance of oxygenic photosynthesis resulted in the recovery of the Shuram excursion. Secular changes in δ^13^C and δ^15^N chemostratigraphy and Raman structural order are useful tools to understand the evolution of late Ediacaran environmental conditions and provide an explanation for the recovery from the most negative carbon isotope excursion in Earth’s history.

## Supplementary information

Supplemetary Information

Description of Additional Supplementary Files

Supplementary data 1

## Data Availability

The authors declare that the data supporting the findings of this study are available within the paper and its supplementary information files (Supplementary Tables).

## References

[1] Calver CR (2000). Isotope stratigraphy of the Ediacarian (Neoproterozoic III) of the Adelaide Rift Complex, Australia, and the overprint of water column stratification. Precambrian Res.

[2] Jiang G, Kaufman AJ, Christie-Blick N, Zhang S, Wu H (2007). Carbon isotope variability across the Ediacaran Yangtze platform in South China: Implications for a large surface-to-deep ocean δ13C gradient. Earth Planet. Sci. Lett..

[3] Fike DA, Grotzinger JP, Pratt LM, Summons RE (2006). Oxidation of the Ediacaran Ocean. Nature.

[4] Verdel C, Wernicke BP, Bowring SA (2011). The Shuram and subsequent Ediacaran carbon isotope excursions from southwest Laurentia, and implications for environmental stability during the metazoan radiation. Bull. Geol. Soc. Am..

[5] McFadden KA (2008). Pulsed oxidation and biological evolution in the Ediacaran Doushantuo Formation. Proc. Natl Acad. Sci..

[6] Grotzinger JP, Fike DA, Fischer WW (2011). Enigmatic origin of the largest-known carbon isotope excursion in Earth’s history. Nat. Geosci..

[7] Li C (2017). Uncovering the spatial heterogeneity of Ediacaran carbon cycling. Geobiology.

[8] Shields GA (2019). Unique Neoproterozoic carbon isotope excursions sustained by coupled evaporite dissolution and pyrite burial. Nat. Geosci..

[9] Cui H, Kaufman AJ, Xiao S, Zhou C, Liu XM (2017). Was the Ediacaran Shuram Excursion a globally synchronized early diagenetic event? Insights from methane-derived authigenic carbonates in the uppermost Doushantuo Formation, South China. Chem. Geol..

[10] Derry LA, Kaufman AJ, Jacobsen SB (1992). Sedimentary cycling and environmental- change in the Late Proterozoic — evidence from stable and radiogenic isotopes. Geochim. Cosmochim. Acta.

[11] Schrag DP (2013). Authigenic carbonate and the history of the global carbon cycle. Science.

[12] Rothman DH, Hayes JM, Summons RE (2003). Dynamics of the Neoproterozoic carbon cycle. Proc. Natl Acad. Sci. USA.

[13] Jiang G (2012). The origin of decoupled carbonate and organic carbon isotope signatures in the early Cambrian (ca. 542 – 520 Ma) Yangtze platform. Earth Planet. Sci. Lett..

[14] Lee C, Love GD, Fischer WW, Grotzinger JP, Halverson GP (2015). Marine organic matter cycling during the Ediacaran Shuram excursion. Geology.

[15] She Z, Strother P, Papineau D (2014). Terminal Proterozoic cyanobacterial blooms and phosphogenesis documented by the Doushantuo granular phosphorites II: Microbial diversity and C isotopes. Precambrian Res.

[16] Ader M (2009). A multilayered water column in the Ediacaran Yangtze platform? Insights from carbonate and organic matter paired δ13C. Earth Planet. Sci. Lett..

[17] Cui H (2016). Phosphogenesis associated with the Shuram Excursion: Petrographic and geochemical observations from the Ediacaran Doushantuo Formation of South China. Sediment. Geol..

[18] Gao Y, Zhang X, Zhang G, Chen K, Shen Y (2018). Ediacaran negative C-isotopic excursions associated with phosphogenic events: Evidence from South China. Precambrian Res.

[19] Scott C (2008). Tracing the stepwise oxygenation of the Proterozoic ocean. Nature.

[20] Sahoo SK (2016). Oceanic oxygenation events in the anoxic Ediacaran ocean. Geobiology.

[21] Ostrander CM (2019). Multiple negative molybdenum isotope excursions in the Doushantuo Formation (South China) fingerprint complex redox-related processes in the Ediacaran Nanhua Basin. Geochim. Cosmochim. Acta.

[22] Shi W, Li C, Algeo TJ (2017). Quantitative model evaluation of organic carbon oxidation hypotheses for the Ediacaran Shuram carbon isotopic excursion. Sci. China Earth Sci..

[23] Li C (2010). A Stratified Redox Model for the Ediacaran Ocean. Science.

[24] Papineau D, Yin J, Devine KG, Liu D, She Z (2021). Chemically Oscillating Reactions during the Diagenetic Formation of Ediacaran Siliceous and Carbonate Botryoids. Minerals.

[25] Jiang G, Shi X, Zhang S, Wang Y, Xiao S (2011). Stratigraphy and paleogeography of the Ediacaran Doushantuo Formation (ca. 635–551Ma) in South China. Gondwana Res.

[26] Kikumoto R (2014). Nitrogen isotope chemostratigraphy of the Ediacaran and Early Cambrian platform sequence at Three Gorges, South China. Gondwana Res.

[27] Lu M (2013). The DOUNCE event at the top of the Ediacaran Doushantuo Formation, South China: Broad stratigraphic occurrence and non-diagenetic origin. Precambrian Res.

[28] Furuyama S (2017). Chemostratigraphy of the Ediacaran basinal setting on the Yangtze platform, South China: Oceanographic and diagenetic aspects of the carbon isotopic depth gradient. Isl. Arc.

[29] Wang X, Jiang G, Shi X, Xiao S (2016). Paired carbonate and organic carbon isotope variations of the Ediacaran Doushantuo Formation from an upper slope section at Siduping, South China. Precambrian Res.

[30] Wang W (2017). Integrated carbon, sulfur, and nitrogen isotope chemostratigraphy of the Ediacaran Lantian Formation in South China: Spatial gradient, ocean redox oscillation, and fossil distribution. Geobiology.

[31] Ader M (2014). Ocean redox structure across the Late Neoproterozoic Oxygenation Event: A nitrogen isotope perspective. Earth Planet. Sci. Lett..

[32] Beaumont V, Robert F (1999). Nitrogen isotope ratios of kerogens in Precambrian cherts: A record of the evolution of atmosphere chemistry?. Precambrian Res.

[33] Redfield AC, Ketchum BH, Richards FA (1963). The influence of organisms on the composition of sea-water. Sea.

[34] Xiao S (2012). Integrated chemostratigraphy of the Doushantuo Formation at the northern Xiaofenghe section (Yangtze Gorges, South China) and its implication for Ediacaran stratigraphic correlation and ocean redox models. Precambrian Res.

[35] Cui H (2015). Redox architecture of an Ediacaran ocean margin: Integrated chemostratigraphic (δ^13^C-δ^34^S-^87^Sr/^86^Sr-Ce/Ce*) correlation of the Doushantuo Formation, South China. Chem. Geol..

[36] Papineau D (2010). Global biogeochemical changes at both ends of the proterozoic: insights from phosphorites. Astrobiology.

[37] Ader M (2016). Interpretation of the nitrogen isotopic composition of Precambrian sedimentary rocks: Assumptions and perspectives. Chem. Geol..

[38] Sigman, D. M., Karsh, K. L. & Casciotti, K. L. Nitrogen isotopes in the ocean. *Encycl. Ocean Sci*. 320–322 (2009).

[39] Mariotti A (1981). Experimental determination of nitrogen kinetic isotope fractionation: Some principles; illustration for the denitrification and nitrification processes. Plant Soil.

[40] Wang J, Chen D, Yan D, Wei H, Xiang L (2012). Evolution from an anoxic to oxic deep ocean during the Ediacaran-Cambrian transition and implications for bioradiation. Chem. Geol..

[41] Lan Z (2019). An integrated chemostratigraphic (δ13C-δ18O-87Sr/86Sr-δ15N) study of the Doushantuo Formation in western Hubei Province, South China. Precambrian Res.

[42] Laakso TA, Sperling EA, Johnston DT, Knoll AH (2020). Ediacaran reorganization of the marine phosphorus cycle. Proc. Natl Acad. Sci..

[43] Lenton TM, Watson AJ (2000). Regulation of nitrate, phosphate, and oxygen in the Ocean. Regulation.

[44] Lee C (2013). Carbon isotopes and lipid biomarkers from organic-rich facies of the Shuram Formation, Sultanate of Oman. Geobiology.

[45] Tyrrell T (1999). The relative influences of nitrogen and phosphorus on oceanic primary production. Nature.

[46] Bronk DA, Gilbert PM, Malone TC, Banahan S, Sahlsten E (1998). Inorganic and organic nitrogen cycling in Chesapeake Bay: Autotrophic versus heterotrophic processes and relationships to carbon flux. Aquat. Microb. Ecol..

[47] Kepkay PE, Jellett JF, Niven SEH (1997). Respiration and the carbon-to-nitrogen ratio of a phytoplankton bloom. Mar. Ecol. Prog. Ser..

[48] Wang H (2016). Depositional environment and micropore characteristics of the Ediacaran Doushantuo Formation black shale in Western Hubei. China Arab. J. Geosci..

[49] Hansell DA, Carlson CA, Repeta DJ, Schlitzer R (2009). Dissolved organic matter in the ocean a controversy stimulates new insights. Oceanography.

[50] Clayton C (1991). Carbon isotope fractionation during natural gas generation from kerogen. Mar. Pet. Geol..

[51] Barber RT (1968). Dissolved Organic Carbon from Deep Waters resists Microbial Oxidation. Nature.

[52] Wang L, Shi X, Jiang G (2012). Pyrite morphology and redox fluctuations recorded in the Ediacaran Doushantuo Formation. Palaeogeogr. Palaeoclimatol. Palaeoecol..

[53] Mulder A, van de Graaf AA, Robertson LA, Kuenen JG (1995). Anaerobic ammonium oxidation discovered in a denitrifying fluidized bed reactor. FEMS Microbiol. Ecol..

[54] Marin-Carbonne J, Robert F, Chaussidon M (2014). The silicon and oxygen isotope compositions of Precambrian cherts: A record of oceanic paleo-temperatures?. Precambrian Res.

[55] Robert F, Chaussidon M (2006). A palaeotemperature curve for the Precambrian oceans based on silicon isotopes in cherts. Nature.

[56] Dodd MS (2019). Widespread occurrences of variably crystalline 13 C-depleted graphitic carbon in banded iron formations. Earth Planet. Sci. Lett..

[57] Foucher F, Ammar MR, Westall F (2015). Revealing the biotic origin of silicified Precambrian carbonaceous microstructures using Raman spectroscopic mapping, a potential method for the detection of microfossils on Mars. J. Raman Spectrosc..

[58] Qu Y, Engdahl A, Zhu S, Vajda V, McLoughlin N (2015). Ultrastructural Heterogeneity of Carbonaceous Material in Ancient Cherts: Investigating Biosignature Origin and Preservation. Astrobiology.

[59] Qu Y (2017). Carbonaceous biosignatures of diverse chemotrophic microbial communities from chert nodules of the Ediacaran Doushantuo Formation. Precambrian Res.

[60] Lahfid A (2010). Evolution of the Raman spectrum of carbonaceous material in low-grade metasediments of the Glarus Alps (Switzerland). Terra Nov..

[61] Li X, Hayashi J (2006). FT-Raman spectroscopic study of the evolution of char structure during the pyrolysis of a Victorian brown coal. Fuel.

[62] Ferralis N, Matys ED, Knoll AH, Hallmann C, Summons RE (2016). Rapid, direct and non-destructive assessment of fossil organic matter via microRaman spectroscopy. Carbon N. Y.

[63] Ogawa H, Amagai Y, Koike I, Kaiser K, Benner R (2001). Production of refractory dissolved organic matter by bacteria. Science.

[64] Osterholz H (2016). Deciphering associations between dissolved organic molecules and bacterial communities in a pelagic marine system. ISME J..

[65] Moran MA (2016). Deciphering ocean carbon in a changing world. Proc. Natl Acad. Sci. USA.

[66] Jørgensen L (2011). Global trends in the fluorescence characteristics and distribution of marine dissolved organic matter. Mar. Chem..

[67] Canfield DE, Knoll AH, Poulton SW, Narbonne GM, Dunning GR (2020). Carbon isotopes in clastic rocks and the Neoproterozoic carbon cycle. Am. J. Sci..

[68] Cremonese L, Shields-Zhou GA, Struck U, Ling HF, Och LM (2014). Nitrogen and organic carbon isotope stratigraphy of the Yangtze Platform during the Ediacaran-Cambrian transition in South China. Palaeogeogr. Palaeoclimatol. Palaeoecol..

[69] Condon D (2005). U–Pb ages from the Neoproterozoic Doushantuo Formation, China. Science.

[70] Hayes JM, Strauss H, Kaufman AJ (1999). The abundance of 13C in marine organic matter and isotopic fractionation in the global biogeochemical cycle of carbon during the past 800 Ma. Chem. Geol..

