# Supplementary material for: Extensive primary production promoted the recovery of the Ediacaran Shuram excursion
Source: Nat Commun. 2022 Jan 10;13:148. doi: 10.1038/s41467-021-27812-5 (PMC8748710; doi:10.1038/s41467-021-27812-5)
Supplement: Supplementary file 1 — Supplemetary Information [file 41467_2021_27812_MOESM1_ESM.pdf]

Supplementary Notes

Methods

a) Sampling strategy

174 samples were collected from 6 different stratigraphic sections, representing four principal lithologies (limestone, dolostone, black shale and chert). Selected portions of each sample with no veining or weathering alteration were crushed until getting a very fine powder. Approximately 5g of each rock powder were stored in small glass vials for analytical purposes.

b) Isotope ratio mass spectrometry (IRMS)

The isotope analyses were performed at the National Environmental Isotope Facility (NEIF) at the British Geological Survey, Keyworth, UK. For carbonate $\delta^{13}C$ and $\delta^{18}O$ isotope analysis, the material was ground to a fine powder in agate. An aliquot of the powder (c. 10mg) was reacted with anhydrous phosphoric acid in vacuo at 25.2°C. The CO_2 liberated was cryogenically separated from water vapour and collected for analysis. Measurements were made on a VG Optima mass spectrometer after offline extraction of CO_2. $\delta^{13}C$ and $\delta^{18}O$ isotope values are reported as per mil (‰) deviations of the isotopic ratios ($^{13}C/^{12}C$, $^{18}O/^{16}O$) calculated to the Vienna Pee Dee Belemnite (VPDB) scale using procedural laboratory standards calibrated against NBS-19. Overall analytical accuracy for these standards is, on average, better than 0.03‰ for $\delta^{13}C$ and 0.2% $\delta^{18}O$ (both 1SD). Duplicate samples (n=15) reproducibility is 0.05‰ for $\delta^{13}C$ and 0.06% $\delta^{18}O$ (1SD).

The co-occurring sedimentary organic fraction was separated via dissolution in 10% HCl at room temperature overnight. After the reaction, samples were rinsed with deionized water several times until reaching a neutral pH. Once neutralized, samples were dried overnight at 40°C and stored in glass vials. δ^{13}Corg analyses were performed by combustion in a Costech ECS4010 Elemental Analyser (EA) on-line to a VG TripleTrap (plus secondary cryogenic trap) and Optima dual-inlet mass spectrometer. $\delta^{13}C_{org}$ values were calculated with respect to the VPDB standard using a within-run laboratory standard (BROC2), calibrated against NBS-22. Replicate analysis of standards indicated an accuracy of <0.1‰ (1 SD), and analyses of duplicates (n=22) yielded a reproducibility better than 0.1‰ (1 SD). %C and %N analyses

were obtained simultaneously using a calibration against Acetanilide as a procedural standard.

Nitrogen isotope analyses were performed by combustion in a Thermo Finnigan Flash EA (1112 series) on-line to a Delta Plus XL mass spectrometer. $\delta^{15}N$ was calculated with respect to the $\delta^{15}N$ value of air using the BROC2 standard as a within-run laboratory standard calibrated against USGS40 and USGS41. Overall analytical accuracy for these samples is, on average, better than 0.2‰. Replicate analysis of some samples (n=8) indicated a reproducibility of <0.2‰ (1 SD).

c) Inductively coupled plasma-optical emission spectrometer (ICP-OES)

We obtained Ca, Mg, Fe, Sr and Mn abundances with an inductively coupled plasma-optical emission spectrometer (ICP-OES, Model: Varian 720-OES) at the Earth Sciences Geochemistry Laboratory UCL (London). Method parameters include Plasma Power: 1.0 k, Plasma Flow: 15.0 L/Min, Aux Flow: 1.50 L/Min and Neb Flow: 0.9 L/Min. Helium was used as the carrier gas, and Argon was used as the make-up gas, mixed with the carrier gas. The elemental abundances were obtained by calibration of peak intensities of sample solutions with a standard analytical solution.

d) Optical microscopy and Raman spectroscopy

Raman spectra and hyperspectral images of organic matter were obtained in the Geological Spectroscopy Laboratory at UCL with a WITec Confocal Raman Imaging system using a 532 nm laser at up to 1000x magnification. Carbonaceous targets were detected in standard petrological thin sections (30 µm in thickness) using an optical microscope (Olympus BX51) equipped with 4x,10x,20x,50x and 100x objectives. Cosmic ray reduction was applied to all spectra, and their backgrounds fitted to a polynomial function and subtracted.

In this study, we followed two different spectra decomposition and peak calculations: 1) only taking into account the main bands at 1350 and 1600 cm^{-1} to calculate the intensity ratio of 1350 cm^{-1}-band versus 1600 cm^{-1}-band, defined as I-1350/1600; and 2) deconvoluting the spectra range from 1090 to 1700 cm^{-1} using a Lorentzian function into five-band peaks: G-band (~1594 cm^{-1}), D1-band (~1350 cm^{-1}), D2-band (~1620 cm^{-1}), D3-band (~1510 cm^{-1}), and D4-band (~1245 cm^{-1}). After deconvolution, the main key Raman spectral parameters are reported: peak centre position, area, width, full width at half maximum (FWHM) and selected peak area ratios. To evaluate maximum metamorphic temperatures of organic matter from

the Raman spectra we used the geothermometer of reference [1], which uses RA1 = (D1 + D4) ⁄(D1 + D2 + D3 + D4 + G) and RA2 = (D1 + D4) ⁄(D2 + D3 + G) to estimate the variation of area ratio with temperature for low-grade rocks.

Isotopic signatures: Post-depositional analysis

Post-depositional alteration assessment is critical to interpreting the chemostratigraphies of C and N in their depositional environments. This is because metamorphic processes alter isotopic signatures of both C and N[2–4]. At the greenschist metamorphic grade, where chlorite forms between about 300-450°C, isotopic signatures are altered and $\delta^{13}C$ in organic matter values can become more ^{13}C-enriched by typically less than about 1-2‰[2,4]. Likewise, $\delta^{15}N$ values of residual sedimentary N can also increase by 1–2‰ under greenschist facies metamorphism[5]. Elemental abundance plots and Raman spectra of organic matter are used to assess the extent of diagenetic and metamorphic alteration on the studied samples.

a) Geochemical cross-plots

The extent of diagenetic and metamorphic alteration on the studied samples was evaluated using linear relationships of elemental abundances in four different plots: $\delta^{13}C_{org}$-TOC, TOC-TN, TN-$\delta^{15}N$ and Mn/Sr- $\delta^{13}C_{carb}$. The $\delta^{13}C_{org}$ -TOC plot is generally used to assess whether the degradation of organic matter during diagenesis affects the $\delta^{13}C_{org}$ data[6]. This is because degradation of organic matter during diagenesis would have caused ^{13}C-depleted loss and residues to be enriched in ^{13}C[2,4]. However, the data from the newly studied samples, shown in Figure S3-a, present no negative correlation between $\delta^{13}C_{org}$ and TOC values, indicating insignificant post-depositional effects $\delta^{13}C_{org}$ values. The TOC-TN plot is usually used to assess the co-variations of nitrogen content with TOC and whether N is derived from organic matter or sourced from inorganic clay-bound nitrogen. In Figure S3-b, it is observed that total nitrogen positively correlates with total organic carbon in the six sections showing a linear relationship with high correlation coefficients ($R > 90$) in most sections, except in Taoying and Fengtan. The linear relationship intercepts on the N axis in these latter sections, meaning that clay-bound nitrogen contributes to the measured N data only in these sections. Thus, it can be inferred that, in general, N mainly occurs in organic matter, and an insignificant isotopic fractionation by diagenesis or metamorphism is expected. The TN-$\delta^{15}N$ plot is generally used to assess whether the loss of N may have substantially altered the $\delta^{15}N$ values. This is because $\delta^{15}N_{bulk}$ values in sediments typically increase with decreasing TN content due

to the preferential loss of ^{14}N during thermal alteration[7]. However, such a negative correlation is not found in the new data, indicating an insignificant alteration of $\delta^{15}N$ values and minimal N loss (Fig. S3-c). Finally, the Mn/Sr ratio is used to evaluate the possible impact of meteoric diagenesis on carbonate. The interaction of carbonates with meteoric fluids during diagenesis, metamorphism, or dolomitization increases the Mn/Sr ratio and decreases the $\delta^{13}C_{carb}$[8]. This is because Sr leaches from carbonate during meteoritic diagenetic processes, and Mn is incorporated in diagenetic cement. It was suggested that carbonates with Mn/Sr <10 usually retain near primary $\delta^{13}C_{carb}$ abundances[9]. The Mn/Sr- $\delta^{13}C_{carb}$ plot in Figure S3-d shows that most of the measured Mn/Sr ratios in this study are <10, and no considerable decrease in $\delta^{13}C_{carb}$ is seen. Thus, meteoric diagenesis on carbonate samples is negligible. Consequently, combined results from the $\delta^{13}C_{org}$-TOC, TOC-TN, TN-$\delta^{15}N$ and Mn/Sr- $\delta^{13}C_{carb}$ cross-plots indicates that alteration of isotopic compositions have not been identified in the studied samples, and therefore, $\delta^{13}C_{carb}$, $\delta^{13}C_{org}$ and $\delta^{15}N_{sed}$ values reflect primary compositions.

b) Raman Spectroscopy

The metamorphic grade is determined using the empirical geothermometer based on organic matter in pelitic sediments from Lahfid *et al.* (2010)[1]. Seventeen representative samples selected from proximal and distal depositional environments are investigated. Figure S4 shows examples of the fitted curves after spectra deconvolution in proximal and distal sections. Two different area ratios, RA1 and RA2, are calculated with a result of 0.60-0.62 and 1.50-1.65, respectively. Results show peak metamorphic temperature (T1) estimates between 280°C and 309°C (Table S2), approximately at the sub-greenschist metamorphic facies and point to insignificant isotopic fractionation. These temperatures are consistent with previous temperatures reported for the Doushantuo Fm.[10,11]. In addition, these results also show a thermal basin distribution with temperatures 280-289 ^{o}C in the proximal sections 300-309 ^{o}C in the distal sections.

Member IV stratigraphic correlation

One of the principal uncertainties in the Nanhua basin is the stratigraphic correlation of Member IV of the Doushantuo Fm. among sections. The Member IV variable thickness throughout the basin, between 1 and 30 m, is thought to be related to the compensation of the rifting-inherited palaeobathymetry, which determined the basin configuration and facies

distribution[12]. Despite Member IV being a laterally continuous organic-rich shale marker bed tracked throughout the basin, the presence of two black shale layers separated by a dolostone unit in the shelf lagoon or the distinct $\delta^{13}C_{carb}$ and $\delta^{13}C_{org}$ profiles in the deep basin raises the possibility of different stratigraphic correlations. Understanding the basin stratigraphy and correlation is highly relevant as, conventionally, the Member IV of the Doushantuo Fm. has been considered time equivalent with the Shuram excursion[13,14], where the upper limit of the excursion can be set at 551.1 Ma[15]. We approach the Member IV correlation problem by comparing stratigraphic and geochemical data from the proximal and distal sections and evaluating the Shuram excursion implications.

Several sections located in the western region of the Yangtze Gorges area (shelf lagoon) present two black shale layers separated by a dolostone interval, named upper black shale (Miaohe Member), upper dolostone (UD) and lower black shale (LBS), respectively[16]. However, it is uncertain how the Miaohe Member, UD and LBS are correlated with Member IV in the rest of the Yangtze Gorges area (central and eastern regions) and distal environments, where Member IV is characterized by a single black shale layer. The uncertainty about the stratigraphic correlation has brought different interpretations whose principal difference lies in the age constraints of the Miaohe biota and, by extension, the duration and age of the DOUshantuo Negative Carbon Excursion (DOUNCE), potentially correlated with the Shuram excursion.

Several stratigraphic correlations have been proposed, such as the “Z” correlation, which suggests a conventional correlation in which the Miaohe Member, UD and LBS are equivalent to Member IV[16,17]. Then, the “A” correlation proposes that the Miaohe Member correlates with the overlying Shibantan Member (Dengying Fm.)[18] rather than Member IV of the Doushantuo Fm. Finally, a third correlation considers the UD interval to be an allochthonous olistostrome due to large and small-scale slumping or faulting, in which case the two shale layers are from the same unit and equivalent to Member IV[19,20].

In our stratigraphic correlation, we use stratigraphic, paleontological and geochemical arguments to support the “Z” correlation for Member IV. The segmented geometry on the Yangtze platform during deposition of the Doushantuo Fm. likely favoured abrupt lateral variation of facies by which the UD interval would have thinned down and pinched out, to a point where it eventually vanished even over a short distance (< 50 km)[12]. Also, taxonomic differences, principally derived from morphological traits, have been reported between the

Miaohe Member and Shibantan Member (Dengying Fm.) biotas[21,22]. Moreover, the Eoandromeda taxa, present in the Miaohe biota, has also been found in Australia, suggesting that the Miaohe biota forms part of the 'White Sea assemblage'[23] whereas the assemblage of fossils in the Shibantan biota is typical of the younger Nama assemblage[22]. In terms of geochemical differences, it has been widely reported that Member IV and the Dengying Fm. have distinct ^{13}C-depletions in organic matter[13,24,25]. Member IV generally shows $\delta^{13}C_{org}$ values between −40‰ and −35‰, whereas the Dengying Fm. shows $\delta^{13}C_{org}$ values between −30‰ and −25‰. In our study, $\delta^{13}C_{org}$ results of the black shales deposited in proximal environments describe similar patterns to those of Member IV rather than the Shibantan Member with values between −38.6‰ and −35.7‰.

However, correlations of Member IV in the distal environments (slope and basin) are less well-constrained due to the limited number of published sections with Member IV and Liuchapo Fm. (basinal equivalent of the Dengying Fm.) data. Previous correlations between proximal and distal sections can help identify chemostratigraphic similarities and locate the base and top of Member IV. For example, the Siduping section, located in the upper slope environment, and the Fengtan section, located in the basin, have been previously correlated with the Jiulongwan section[26,27]. In these correlations, the two negative $\delta^{13}C_{carb}$ excursions (e.g. N1 and N2) before the Shuram anomaly are correlated. Then, the base of the Shuram anomaly is tracked following the same criteria as in the Yangtze Gorges area, that is, the shift towards negative $\delta^{13}C_{carb}$ values in Member III. However, the top of the anomaly is marked by the base of the chert unit corresponding to the overlying Dengying Fm./Liuchapo Fm. This boundary between the top of Member IV and the base of Dengying Fm./Liuchapo Fm. serves as a stratigraphic marker to identify the different $\delta^{13}C_{carb}$ and $\delta^{13}C_{org}$ trends in other distal sections. For example, in contrast to $\delta^{13}C_{carb}$ values of the Dengying Fm., which vary from ∼ −4‰ to +4‰ in proximal environments, $\delta^{13}C_{carb}$ values of the Liuchapo Fm. range from ∼ −5‰ to −10‰ in distal environments. Similarly, $\delta^{13}C_{org}$ of the Dengying Fm. varies from ∼ −39‰ to −30‰, in contrast to $\delta^{13}C_{org}$ of the Liuchapo Fm. that varies ∼ −33‰ to ∼ −37‰ in the slope or remains stable ∼ −33‰ in the basin.

The new geochemical data seem to follow robust similarities with the "Z" correlation in the proximal paleo-environments. At the same time, the new $\delta^{13}C_{org}$ values allowed us to identify chemostratigraphic similarities and locate the base and top of Member IV of the distal sections. Figure S2 shows the Nanhua basin stratigraphic correlation where the black shales

described in this study correlate with the Member IV of the Doushantuo Fm. and are interpreted to be equivalent to the Shuram excursion.

References

1. Lahfid, A. *et al.* Evolution of the Raman spectrum of carbonaceous material in low-grade metasediments of the Glarus Alps (Switzerland). *Terra Nov.* **22**, 354–360 (2010).
2. Freudenthal, T., Wagner, T., Wenzhöfer, F., Zabel, M. & Wefer, G. Early diagenesis of organic matter from sediments of the Eastern subtropical Atlantic: Evidence from stable nitrogen and carbon isotopes. *Geochim. Cosmochim. Acta* **65**, 1795–1808 (2001).
3. Bebout, G. E. & Fogel, M. L. Nitrogen-isotope compositions of metasedimentary rocks in the Catalina Schist, California: Implications for metamorphic devolatilization history. *Geochim. Cosmochim. Acta* **56**, 2839–2849 (1992).
4. Lehmann, M. F., Bernasconi, S. M., Barbieri, A. & McKenzie, J. A. Preservation of organic matter and alteration of its carbon and nitrogen isotope composition during simulated and in situ early sedimentary diagenesis. *Geochim. Cosmochim. Acta* **66**, 3573–3584 (2002).
5. Ader, M. *et al.* Interpretation of the nitrogen isotopic composition of Precambrian sedimentary rocks: Assumptions and perspectives. *Chem. Geol.* **429**, 93–110 (2016).
6. Hayes, J., Wedeking, K. & Kaplan, I. Precambrian organic geochemistry - Preservation of the record. in (1983).
7. Minagawa, M. & Wada, E. Nitrogen isotope ratios of red tide organisms in the East China Sea: A characterization of biological nitrogen fixation. *Mar. Chem.* **19**, 245–259 (1986).
8. Brand, U. & Veizer, J. Chemical diagenesis of a multicomponent carbonate system - 2: stable isotopes. *J. Sediment. Petrol.* **51**, 987–998 (1981).
9. Kaufman, A. J. & Knoll, A. H. Neoproterozoic variations in the C-isotopic composition of seawater: stratigraphic and biogeochemical implications. *Precambrian Res.* **73**, 27–49 (1995).
10. Wang, Z. *et al.* Raman geothermometry of carbonaceous material in the basal Ediacaran Doushantuo cap dolostone: The thermal history of extremely negative δ13C signatures in the aftermath of the terminal Cryogenian snowball Earth glaciation. *Precambrian Res.* **298**, 174–186 (2017).
11. Chang, B. *et al.* Massive formation of early diagenetic dolomite in the Ediacaran ocean: Constraints on the 'dolomite problem'. *Proc. Natl. Acad. Sci. U. S. A.* **117**, 14005–14014 (2020).
12. Vernhet, E. Paleobathymetric influence on the development of the late Ediacaran Yangtze platform (Hubei, Hunan, and Guizhou provinces, China). *Sediment. Geol.* **197**, 29–46 (2007).
13. McFadden, K. A. *et al.* Pulsed oxidation and biological evolution in the Ediacaran Doushantuo Formation. *Proc. Natl. Acad. Sci.* **105 (9)**, 3197–3202 (2008).
14. Zhu, M., Zhang, J. & Yang, A. Integrated Ediacaran (Sinian) chronostratigraphy of South China. *Palaeogeogr. Palaeoclimatol. Palaeoecol.* **254**, 7–61 (2007).
15. Condon, D. *et al.* U–Pb ages from the Neoproterozoic Doushantuo Formation, China. *Science.* **308**, 95–98 (2005).
16. Zhou, C. *et al.* The stratigraphic complexity of the middle Ediacaran carbon isotopic

record in the Yangtze Gorges area, South China, and its implications for the age and chemostratigraphic significance of the Shuram excursion. *Precambrian Res.* **288**, 23–38 (2017).
17. Xiao, S., Bykova, N., Kovalick, A. & Gill, B. C. Stable carbon isotopes of sedimentary kerogens and carbonaceous macrofossils from the Ediacaran Miaohe Member in South China: Implications for stratigraphic correlation and sources of sedimentary organic carbon. *Precambrian Res.* **302**, 171–179 (2017).
18. An, Z. *et al.* Stratigraphic position of the Ediacaran Miaohe biota and its constrains on the age of the upper Doushantuo δ13C anomaly in the Yangtze Gorges area, South China. *Precambrian Res.* **271**, 243–253 (2015).
19. Lu, M. *et al.* The DOUNCE event at the top of the Ediacaran Doushantuo Formation, South China: Broad stratigraphic occurrence and non-diagenetic origin. *Precambrian Res.* **225**, 86–109 (2013).
20. Zhu, M. M. Y. *et al.* Carbon isotope chemostratigraphy and sedimentary facies evolution of the Ediacaran Doushantuo Formation in western Hubei, South China. *Precambrian Res.* **225**, 7–28 (2013).
21. Xiao, S., Yuan, X., Steiner, M. & Knoll, A. H. Macroscopic carbonaceous compressions in a terminal Proterozoic shale: a systematic reassessment of the Miaohe Biota, South China. *South China J. Paleontol.* **76**, 347–376 (2002).
22. Chen, Z. *et al.* New Ediacara fossils preserved in marine limestone and their ecological implications. *Sci. Rep.* **4**, 1–10 (2014).
23. Zhu, M., Gehling, J. G., Xiao, S., Zhao, Y. & Droser, M. L. Eight-armed Ediacara fossil preserved in contrasting taphonomic windows from China and Australia. *Geology* **36**, 867–870 (2008).
24. Kikumoto, R. *et al.* Nitrogen isotope chemostratigraphy of the Ediacaran and Early Cambrian platform sequence at Three Gorges, South China. *Gondwana Res.* **25**, 1057–1069 (2014).
25. Wang, X. Q., Shi, X. Y., Jiang, G. Q. & Tang, D. J. Organic carbon isotope gradient and ocean stratification across the late Ediacaran-Early Cambrian Yangtze Platform. *Sci. China Earth Sci.* **57**, 919–929 (2014).
26. Wang, X., Jiang, G., Shi, X. & Xiao, S. Paired carbonate and organic carbon isotope variations of the Ediacaran Doushantuo Formation from an upper slope section at Siduping, South China. *Precambrian Res.* **273**, 53–66 (2016).
27. Furuyama, S. *et al.* Chemostratigraphy of the Ediacaran basinal setting on the Yangtze platform, South China: Oceanographic and diagenetic aspects of the carbon isotopic depth gradient. *Isl. Arc* **26**, 1–14 (2017).

Supplementary Figures

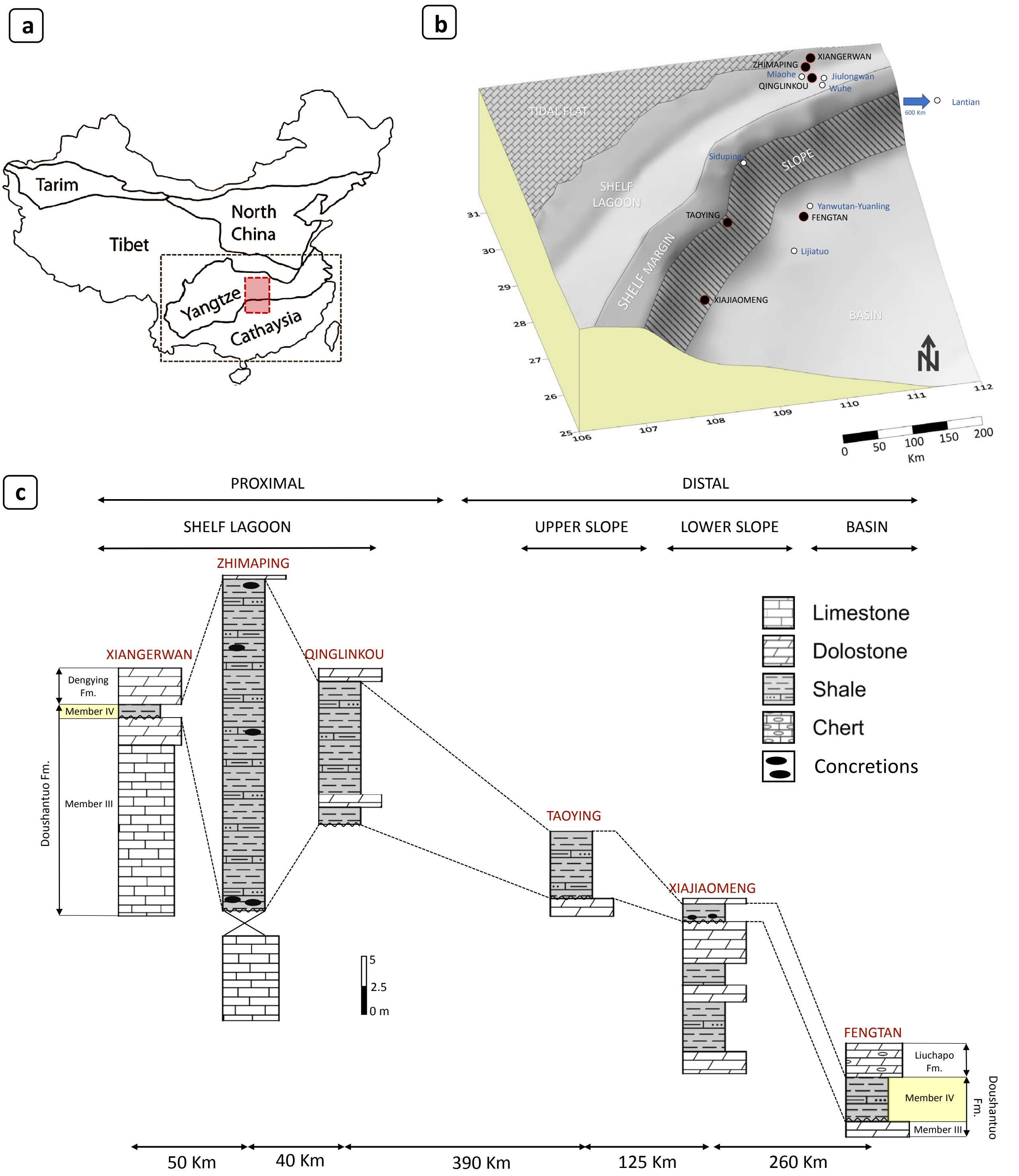

Fig. S1 a Locality map of the South China block and the Ediacaran Yangtze platform. The dashed square indicates the Nanhua basin approximate extension. **b** Basin-scale paleogeographic reconstruction during the deposition of the Ediacaran Doushantuo Fm.[1]. Black circles represent the studied sections. White circles represent sections from the literature used to complement this study. **c** Simplified lithostratigraphic column and Member IV correlation of the six studied sections.

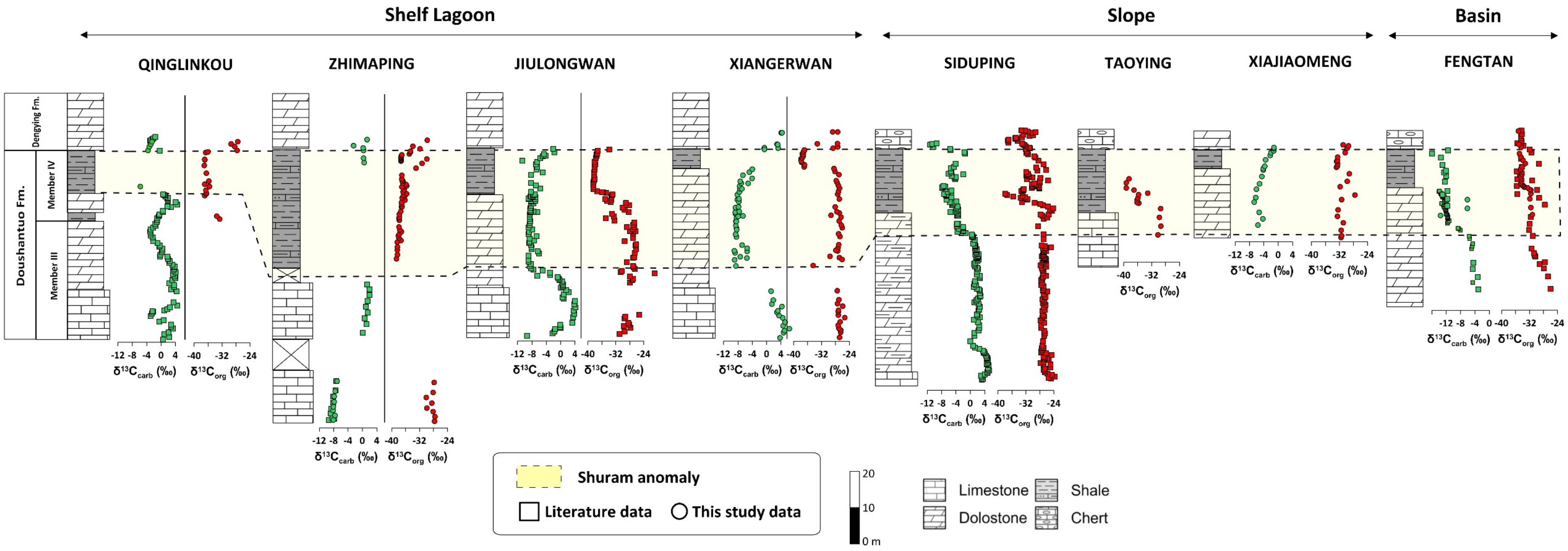

Fig. S2 Proposed stratigraphic correlation of the Shuram anomaly. The location of the sections is shown in Figure S1b. The correlation of Member IV in the shelf lagoon is the same as the "Z" correlation[3], where the Miaohe Member in the western region (Qinglinkou) correlates with the single black shale unit in the central region (Jiulongwan). $\delta^{13}C_{carb}$ and $\delta^{13}C_{org}$ data from this study, Jiulongwan[4], Siduping[5] and Fengtan [This study, 6,7].

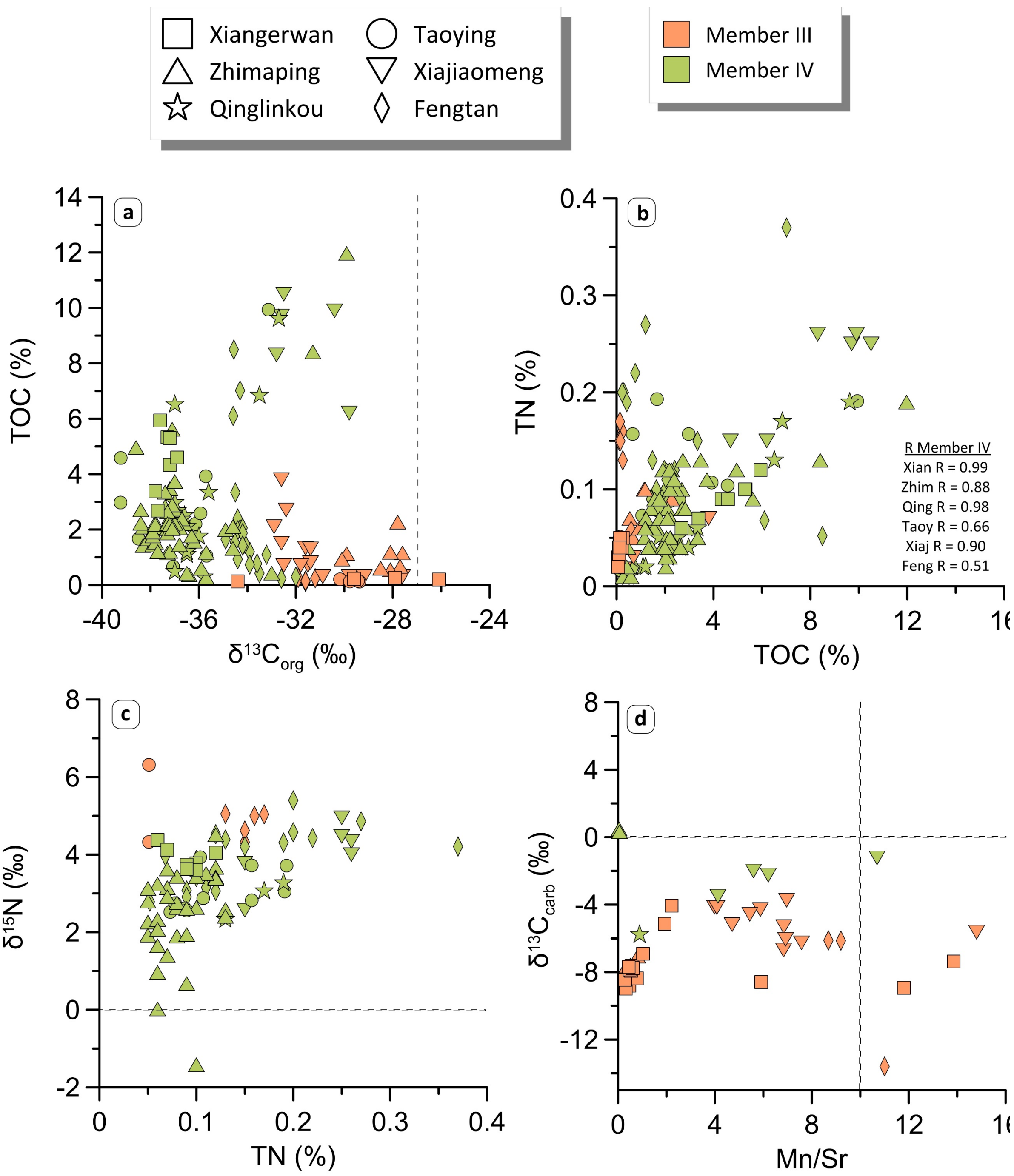

Fig. S3 Measured isotopic (C and N) compositions and contents (C, N and Mn) plots. a $\delta^{13}C_{carb}$ vs TOC, **b** TOC vs TN, **c** TN vs $\delta^{15}N$, and **d** Mn/Sr versus $\delta^{13}C_{carb}$. Correlation coefficients (R) in **b** correspond to Member IV. The vertical line in **a** corresponds to the isotopic fractionation imparted to biomass by primary producers typically around −27‰. The horizontal line in **c** corresponds to $\delta^{15}N$ reference standard 0‰ (Atmospheric N_2). The vertical line in **d** corresponds to the meteoric alteration Mn/Sr threshold below which primary $\delta^{13}C_{carb}$ abundances are retained[8]. The horizontal line in **d** corresponds to $\delta^{13}C_{carb}$ reference standard 0‰ (Vienna Peedee belemnite).

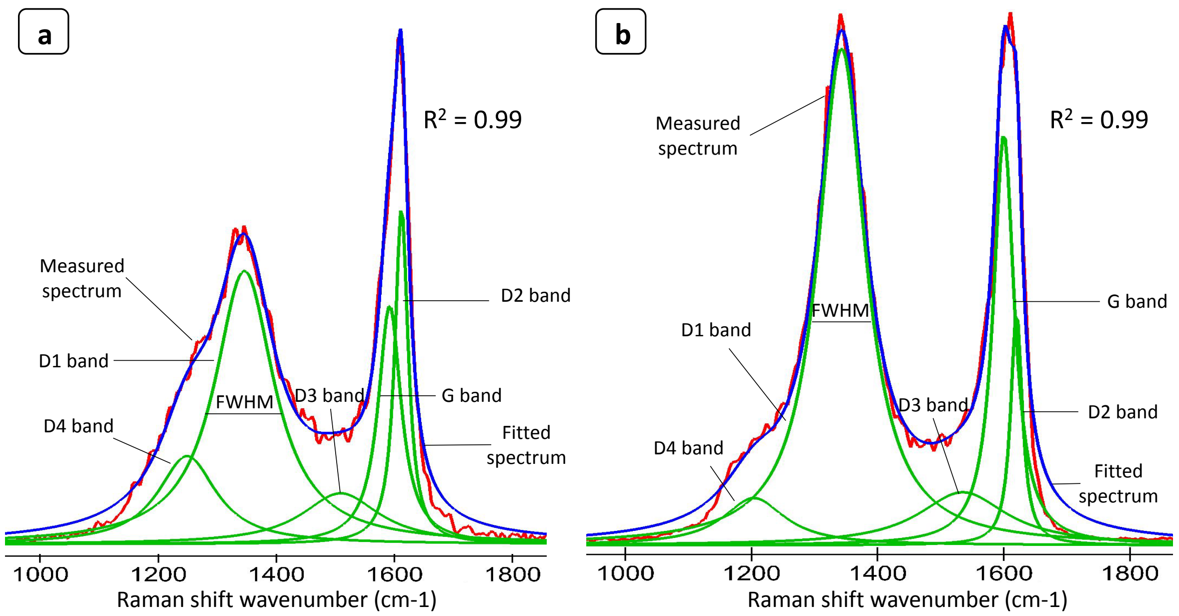

Fig. S4 Lorentzian peak-fitting of the Raman spectrum for two organic-rich samples. The two first-order Raman bands of organic matter at ~1350 cm^{-1} and ~1600 cm^{-1} are represented. **a** Proximal environment (Qinglinkou section). **b** Distal environment (Fengtan section). Peak decomposition in bands (G, D1, D2, D3 and D4) and the parameter FWHM (full width at half maximum) are shown. The coefficient of variation (R^2) compares the original spectrum (red) with the fitted Raman spectrum (blue).

Supplementary Table

Table S2

SAMPLE	G			D1			D2			D3			D4			R^2	RA1	RA2	T1 (°C)	T2 (°C)	Intensity	
	Position	FWHM	Area	Position	FWHM	Area	Position	FWHM	Area	Position	FWHM	Area	Position	FWHM	Area						I-1350	I-1600
**XEW-ZY 10**	1594	50	4,695	1349	136	12,500	1613	31	2,172	1512	180	3,200	1242	130	2,800	0.99	0.60	1.52	284	278	70	97
**XEW-ZY 9**	1590	64	6,500	1351	135	14,000	1609	30	2,000	1507	140	3,250	1245	140	3,900	0.98	0.60	1.52	285	279	144	203
**XEW-ZY 6**	1590	51	4,138	1341	135	10,500	1612	26	1,486	1508	150	2,400	1241	120	1,800	0.98	0.61	1.53	287	281	34	49
**ZMP 13C**	1585	72	4,800	1360	133	8,500	1620	40	600	1510	160	2,600	1248	155	3,500	0.98	0.60	1.50	280	273	135	204
**F13**	1594	60	25,000	1344	113	90,000	1611	35	24,000	1517	160	21,000	1246	120	15,800	0.98	0.60	1.51	283	276	117	167
**F3**	1596	55	2,000	1350	125	5,800	1615	30	1,237	1505	160	1,200	1248	100	900	0.98	0.60	1.51	282	276	66	98
**QLK 18**	1590	59	2,173	1350	147	4,900	1611	24	646	1513	180	1,400	1245	150	1,582	0.99	0.61	1.54	287	281	65	91
**QLK 16**	1592	62	11,000	1348	160	25,500	1612	23	2,100	1520	160	5,800	1240	110	3,500	0.99	0.61	1.53	287	281	304	470
**QLK 9**	1592	47	4,144	1346	100	12,300	1612	27	3,362	1509	160	3,000	1249	120	3,900	0.99	0.61	1.54	289	283	300	450
**18-TY-10**	1592	48	3,500	1344	120	11,700	1609	40	2,400	1515	170	2,800	1246	130	2,000	0.99	0.61	1.57	295	290	79	99
**18-TY-12**	1594	40	5,600	1343	118	41,600	1607	45	15,500	1514	180	9,000	1242	127	5,500	0.98	0.61	1.56	293	288	49	60
**XJMW 37**	1590	49	1,387	1348	134	4,038	1611	30	946	1505	150	814	1249	120	1,000	0.99	0.62	1.60	300	296	22	33
**XJMW 41**	1590	63	13,800	1347	157	45,700	1606	39	9,800	1505	160	7,500	1240	110	4,000	0.98	0.62	1.60	299	295	126	198
**XJMW 45**	1591	33	4,548	1351	156	45,566	1603	42	17,098	1510	137	10,800	1245	110	7,020	0.99	0.62	1.62	303	300	204	328
**FT 0.12**	1600	40	7,000	1344	88	19,500	1620	23	2,364	1530	160	4,300	1204	120	3,100	0.99	0.62	1.65	309	308	48	46
**FT 0.4**	1600	45	3,900	1341	100	10,200	1618	25	250	1535	170	3,000	1206	121	1,476	0.98	0.62	1.63	306	303	113	109
**FT 0.8**	1600	40	6,000	1343	94	18,000	1620	24	2,600	1530	160	4,600	1204	141	3,800	0.99	0.62	1.65	309	307	66	64

Parameters of Raman spectra obtained from the decomposition of 17 carbonaceous samples from Member IV of the Doushantuo Fm. FWHM= full width at half maximum. R^2=coefficient of variation that compares the original spectrum with the fitted Raman spectrum. RA1, RA2, T1 and T2 calculated from Lafhid *et al*. (2004). RA1 = (D1 + D4) ⁄ (D1 + D2 + D3 + D4 + G); RA2 = (D1 + D4) ⁄ (D2 + D3 + G), T1=(RA1-0.3758)/0.0008; T2=(RA2-0.27)/0.0045. I-1350 and I-1600 correspond to the intensities of the 1350 and 1600 cm^{-1} bands.
