# Supplementary material for: Extensive primary production promoted the recovery of the Ediacaran Shuram excursion
Source: Nat Commun. 2022 Jan 10;13:148. doi: 10.1038/s41467-021-27812-5 (PMC8748710; doi:10.1038/s41467-021-27812-5)
Supplement: Supplementary file 2 — Description of Additional Supplementary Files [file 41467_2021_27812_MOESM2_ESM.docx]

Description of Additional Supplementary Files

File name: Supplementary Data 1

Description: Geochemical results obtained from the six studied stratigraphic sections.
